# Continued Failure of Rabies Elimination—Consideration of Challenges in Applying the One Health Approach

**DOI:** 10.3389/fvets.2022.847659

**Published:** 2022-03-29

**Authors:** Siriporn Ghai, Thiravat Hemachudha

**Affiliations:** Thai Red Cross Emerging Infectious Diseases Health Science Centre, King Chulalongkorn Memorial Hospital, Bangkok, Thailand

**Keywords:** rabies, One Health approach, collaboration, disease prevention and control, United Against Rabies

## Introduction

Rabies is a fatal, yet vaccine-preventable neglected zoonotic disease (1); however, control continues to evade public health measures and researchers, whether due to neglect, poor awareness of the disease, limited economic impact, minimal societal pressure to eliminate it, or lack of programmatic funds (2). Rabies infects mammalian species through infected saliva, but dogs are the main reservoirs of rabies. Cost-effective control includes vaccination and population control of the canid reservoir, i.e., prevention at the source. Many parts of the Western Hemisphere were successful in eliminating canine rabies, likely due to mass culling in 19th century and perhaps early 20th century, followed by robust legislation, however rabies continues to be endemic in numerous countries in Asia and Africa (3, 4). As noted by many in the past, human rabies deaths is only an estimate as it remains underreported (5).

One Health is a collaborative, cross-disciplinary effort between public, environmental, and animal health sectors, recognizing that disease outbreaks are multifactorial (6). Integration of disease epidemiology enables a holistic and enduring approach to disease prevention and control in humans. “One Health” was first mentioned during the Severe Acute Respiratory Syndrome (SARS) outbreak in 2003–2004, as a concept that “clearly recognized the link between human and animal health and the threats that diseases pose to food supplies and economies” (7). However, the concept of One Health is not new, and can be traced back at least 200 years (previously known as One Medicine, then One World, One Health) (8).

It is undeniable that rabies elimination can be more effectively achieved by engaging multiple sectors in a One Health collaboration due to the zoonotic and bio-social nature of the disease (9, 10). In addition to canine vaccination campaigns, microchipping programmes, and population control, it is important for countries to coordinate multi-sectorally, and integrate their approaches to eliminating rabies. From the in-country research teams' decades-long experiences in rabies and progress tracking, the authors strongly believe that countries need to increase their accountability, directly impacting policymaking and prioritization, in order to unlock access to required funding. Hospitals and clinics need to be equipped (stock and correctly store) and trained to calculate and administer post-exposure prophylactics and immune globulins, as required. Surveillance activities need to continue as it identifies action points, and feeds back on how successful the control measures have been. Rabies, a neglected and underreported disease, should become a notifiable disease, tying in with country ownership and prioritization.

As OIE leads the collective “United Against Rabies” to drive progress toward “Zero human deaths from dog-mediated rabies by 2030” (11), what are the additional considerations to ensure that One Health, and the goal, is achieved? Rabies has indisputable animal origins, with association with dogs since antiquity (12), therefore the human health sector cannot work in solitary. In the One Health approach defined by the WHO Expert Committee on Rabies since 1991, in addition to diagnostics, vaccination campaigns, and post-exposure treatments in both humans and canines, epidemiological surveillance and community participation have been encouraged, as well as legislative actions (13). If neither the elimination approach nor the disease is new, then why is rabies continuing to kill more than 59,000 humans every year? (14). This paper aims to serve as a reminder of the additional challenges, from the author's experiences, that countries need to overcome or address in order to successfully achieve the WHO's goal by 2030.

## Collaboration Failures

Collaboration, although vital, is not easy. Though the issues outlined below are not specific to rabies, it does apply to global rabies elimination effort. Increased globalization, economic and population growth, and technological advancements resulted in increased competition and reduced research funding and resources. Stakeholders continue to be pitted against one another to compete for limited resources rather than encouraged to pool resources toward a common goal (3, 15). The world is used to living in a competitive culture and are worried that pooling resources and taking risks together will result in loss of rewards that they are working to gain. Additionally, they worry that others will take advantage of them, use them, steal credit, or leave them behind. Therefore, it is important to take the time to learn to work together cooperatively, establish trust, and clearly define roles and responsibilities when collaborating (16).

Despite shared aspirations, each organization has a radically different method and approach. It is not always straightforward to identify the root of the problem. For example, the specific facilitating factors for the transmission of rabies in one country often does not apply to another (3). Different sectors bring in different expertise and perspectives. Therefore, it is crucial to spend time identifying the general problems and specific issues, which can help to clarify a shared common goal and develop a mission statement to define direction for the collaboration (16, 17). It is important to frame problems at a higher level, which is relevant to all sectors and for the public good (18), while acknowledging competing interests. By establishing procedural ground rules from the beginning of the collaboration such as how the decisions will be made, who will speak to the media, publications rights, etc., trust is grown and opportunities for mistakes and misunderstandings are reduced (16, 19). Increased trust leads to increased commitment, accommodates the various points of views, as partners grow more comfortable in compromising their own interests and giving up previous organizational requirements. It is also important for partners to accept the loss of autonomy and recognition and to speak with a single voice, inherent to collaboration (17). Additionally, to address the societal issue, cultural and civic norms should be integrated in the approach to engender collaborations unique to each nation's strategy for lasting impact.

## Data Sharing Failures

To effectively identify the specific obstructions in combating rabies in a country, it is vital to coordinate and share data among the human, animal, plant, and environmental health sectors (20), such as systematic data collection and management of dog population surveys, number of suspected and confirmed dog and human rabies cases, human-dog population densities, and vaccination statistics. Data shared may be used for variety of analytical purposes such as holistic examination of the annual epidemiological trends in humans, wild and domestic animals, to better allocate resources to rabies prevention and control activities (21). However, data sharing raises important cultural, ethical, financial, and technical challenges. It is not easy to strike a balance between data accessibility while safeguarding privacy, determining authorship, and protecting intellectual property. Lack of clarity regarding intellectual property and ownership rights often hinders sharing of data, or obscures who has the authority to decide. Technical issues or necessary resources required to manage and transform data for compatibility purposes are also cited as barriers to data sharing, especially in low- and middle- income countries (22). With clearly defined roles and responsibilities for each organization, it is additionally important for the collaborative working group to further define how authorship is determined and how intellectual property will be protected from the very beginning. Without data sharing, One Health approach to eliminating rabies fails at the core.

## Proof of Burden

As many countries are seeing the profits of their investment in the decrease of human rabies cases and deaths to single digits per year, it becomes harder for these countries to justify the required resources to eliminate rabies once and for all, which can be visualized with an asymptote curve. This “asymptotic phenomenon” makes it harder for countries approaching zero human rabies deaths to achieve this milestone. It is even more difficult for the countries to maintain this milestone. One of the contributing factors to the asymptotic phenomena is identified by Miao and team, where the diminution of human rabies deaths is mistakenly labeled as “progress” by the political sector. This leads to the relaxation of subsequent control efforts. Intermittent “relaxation” and commitment of these efforts leads to human rabies “peaks” or “epidemic waves,” as was observed in China, and is currently observed globally with the COVID-19 pandemic (23). The only way to combat this is to maintain pressure and preserve the priority status of the disease at the country level. This has proved to be beneficial in many countries in the past (20). Most recently, India, which accounts for 36% of the world's rabies cases, launched a new National Action Plan which gives priority by raising awareness of the importance of action against rabies and has now declared rabies a notifiable disease. The plan aims to operationalize One Health through better coordination and communication between the animal- and human health and other relevant sectors. If successful, India's plan can help set an example for other rabies endemic countries (24).

## Policy and Legislative Failures

Rabies elimination requires mass vaccination campaigns, increased vaccination coverage and improved efficiency of vaccination. It requires country ownership, regulatory might, policy changes, and capacity building (25, 26). Political will for eliminating rabies needs to be well-established, despite the lack of direct disease burden or due to the aforementioned “asymptotic phenomena.” There needs to be a recognition that no solid quantitative data is available because most human deaths occur at home as the disease is neglected. Additionally, poor sanitary conditions in rural and urban areas favor an increase in roaming dog populations (20). Thus, without prioritization from the country's political sector to implement proper legal framework and allocate funds and resources to regulate vaccination and sterilization of stray and community dogs, and to raise awareness among dog-owners and public on the importance of pre- and post-exposure vaccinations for both dogs and humans and parameters of dos and don'ts, epidemic waves of rabies will continue. Neglecting these epidemic waves could mean expanding geography, because rabies knows no borders, or host-shift, for example new cases have been recorded in previously rabies-free or low-incidence provinces in China (23). In-country teams need to brainstorm innovative ways of seizing their national political sector's attention toward advocating for the need to eliminate rabies. This could be simply by ensuring that politicians are aware of the connection between canine management and human lives (27), engaging local and political leaders to raise awareness of rabies preventive measures and risky behaviors, underlining the unseen burdens, highlighting the impact of previously successful programmes (28), and even using social media's influence to raise awareness.

## COVID-19 Pandemic

COVID-19 has disrupted the lives and economies of most countries all over the world, whether directly or indirectly (29). As public health professionals are diverted to working on the pandemic, it has disrupted vaccination campaigns in countries (30), including rabies vaccination, and field training. Field staff were overloaded with COVID-19 related duties, and rabies was once again neglected (31). Additionally, due to prioritization failure and supply chain disruption, many remote provinces, where rabies is often widespread (5, 12), faced vaccine shortage (31, 32). Due to lockdowns, fewer people were leaving home, and increased difficulty in travel to and from hospitals or clinics led to decrease in identification of suspected rabid dogs, slower dispatch of rabies response teams, and delayed removal of suspected rabid dogs (30). Lockdowns also deterred people from seeking medical attention due to fear of contracting COVID-19 or because hospitals were overflowing. The pandemic exacerbated the tendencies to neglect post-exposure treatment, underscored by lack of awareness of the threats and dangers of rabies. Lastly, sick COVID-19 patients and those economically affected by the pandemic are unable to care for their pets, which has led to an increase in stray dogs. As the world continues to battle the pandemic, true data on the preventable human-rabies cases and deaths will take some time to emerge. As COVID-19 continues to challenge the public health sector, it is important to develop strategies for rabies prevention and surveillance activities which can accommodate social distancing (30), such as oral rabies vaccine for dogs (33), survive lockdowns, and account for and manage abandoned pets.

## Discussion

The goal and approaches to eliminate rabies, a disease with clear transmission dynamics, although well-established, have not been successful to date. It is time to target the political sector, to ensure that temporary disease burden reduction is not misconstrued as progress, to ensure that a legal framework is in place, and that the strategies account for the restrictions imposed by the COVID-19 pandemic. It is crucial that countries maintain pressure and preserve the priority status of the disease at the country level, so that rabies can be eliminated once and for all.

## Author Contributions

SG drafted the manuscript. TH critically reviewed the manuscript. Both authors contributed to the article and approved the submitted version.

## Funding

This work was supported in part by grant from the Thai Red Cross Society-Emerging Infectious Disease-Health Science Center (TRC-EID-HS) provided by benefactors from all over the country.

## Conflict of Interest

The authors declare that the research was conducted in the absence of any commercial or financial relationships that could be construed as a potential conflict of interest.

## Publisher's Note

All claims expressed in this article are solely those of the authors and do not necessarily represent those of their affiliated organizations, or those of the publisher, the editors and the reviewers. Any product that may be evaluated in this article, or claim that may be made by its manufacturer, is not guaranteed or endorsed by the publisher.
